# Ophelia Syndrome: Acute Stupor as a Primary Manifestation of Hodgkin Lymphoma Diagnosed by Fluorodeoxyglucose Positron Emission Tomography

**DOI:** 10.7759/cureus.114019

**Published:** 2026-08-05

**Authors:** Anibal Cortes Bravo, Abel Yurialdy Espinoza Rios, Andres Mauricio Bonilla Navarrete, Camila Andrea Fontecha Delgado, Angel Fabian Leon Chavez, Katherine Rocio Torres Casas

**Affiliations:** 1 Critical Care Medicine, Instituto Nacional de Cancerología, Bogota, COL; 2 Neurology, Instituto Nacional de Cancerología, Bogota, COL; 3 General Medicine, Instituto Nacional de Cancerología, Bogota, COL; 4 Internal Medicine, Universidad El Bosque, Bogota, COL

**Keywords:** hodgkin lymphoma, mglur5, ophelia syndrome, paraneoplastic limbic encephalitis, pns-care score, positron emission tomography, stupor, tiara sign

## Abstract

Ophelia syndrome is a rare form of paraneoplastic limbic encephalitis associated with Hodgkin lymphoma, mediated by autoantibodies targeting the metabotropic glutamate receptor type 5 (mGluR5). It is characterized by a spectrum of neurological and psychiatric manifestations. Impaired consciousness progressing to stupor as the presenting feature is uncommon and, combined with a non-diagnostic structural MRI, can substantially delay diagnosis and treatment. We report a 59-year-old male with recently diagnosed classical Hodgkin lymphoma who developed acute unexplained stupor after recovery from a hospital-acquired pneumonia, prior to any onco-specific treatment. A comprehensive workup, including brain MRI with 3-Tesla epilepsy protocol, cerebrospinal fluid analysis, viral multiplex PCR, flow cytometry, and extensive metabolic and autoimmune panels, was either normal or non-diagnostic. Only low-titer antinuclear antibodies (ANA, 1:80, speckled pattern) were detected. An empirical trial of levetiracetam for suspected non-convulsive status epilepticus produced no response. Given persistent stupor and exhaustive exclusion of alternative etiologies, a fluorodeoxyglucose brain positron emission tomography was performed. It revealed marked bilateral hippocampal hypermetabolism, diffuse cortical hypometabolism with relative metabolic preservation of the primary motor and visual cortices (the “tiara sign”), and hypermetabolism of the basal ganglia, a functional pattern highly consistent with paraneoplastic limbic encephalitis. Integrating these findings with subsequent histopathological confirmation of mixed-cellularity classical Hodgkin lymphoma yielded a Paraneoplastic Neurologic Syndrome (PNS)-Care Score of 7, classifying the case as definite paraneoplastic neurological syndrome. Rescue immunosuppression with methylprednisolone pulses (1 gram per day for three days) led to dramatic neurological recovery within 72 hours, and chemotherapy produced complete resolution of the neurological syndrome. This case highlights that a normal structural MRI does not exclude paraneoplastic limbic encephalitis. When unexplained stupor or coma occurs in an oncological patient with a non-diagnostic conventional workup, molecular functional imaging should be incorporated early into the diagnostic algorithm, as its functional metabolic signature enables timely classification under PNS-Care Score criteria and initiation of immunosuppressive therapy capable of reversing potentially life-threatening neurological deficits.

## Introduction

Paraneoplastic neurological syndromes (PNS) represent a heterogeneous group of immune-mediated disorders resulting from cross-reactive immune responses against central nervous system antigens ectopically expressed by distant neoplasms [1,2]. Although their prevalence is below 1% in the global oncological population, their identification constitutes a first-order clinical imperative: neurological symptoms precede the diagnosis of the primary neoplasm in approximately 80% of cases [2-4]. Among lymphoid malignancies, Hodgkin lymphoma (HL) is recognized as the third most frequent cause of paraneoplastic limbic encephalitis (PLE), following small cell lung carcinoma and germ cell tumors [3,5]. The specific association between HL and PLE is known as Ophelia syndrome, an entity originally described by Carr in 1982, whose eponym evokes the disorientation and cognitive decline of Shakespeare’s character in Hamlet [6-9].

From a neurochemical perspective, glutamate receptors are the primary mediators of excitatory synaptic transmission, neuronal plasticity, and memory consolidation. They are classified as ionotropic glutamate receptors (iGluRs) and metabotropic glutamate receptors (mGluRs), with the latter serving as critical regulators of neuronal excitability [10]. The pathogenesis of Ophelia syndrome rests on the production of autoantibodies directed against the extracellular domain of the metabotropic glutamate receptor type 5 (mGluR5), located predominantly in the postsynaptic terminals of the hippocampus and amygdala [2,5,10,11].

Tumor immunobiology studies suggest this breakdown of self-tolerance is not random, but the result of molecular mimicry: Reed-Sternberg cells in classical HL ectopically express the mGluR5 receptor on their surface [7,12]. This antigenic aberration is amplified by the unique immune microenvironment of HL, conditioned by alterations in chromosome 9p24.1 that increase expression of PD-L1 and PD-L2 ligands, facilitating systemic dysregulation that favors expansion of autoreactive B-cell clones [7,10].

The diagnosis of anti-mGluR5 PLE constitutes a major challenge because of the diversity of its manifestations, including subacute anterograde amnesia, epileptic seizures, and neuropsychiatric changes, and the limited accessibility of antibody testing in serum or cerebrospinal fluid [5,13,14]. Historically, the workup has relied on MRI and electroencephalography; however, structural neuroimaging is normal in 30% to 50% of patients in early phases, leading to therapeutic delays that compromise functional prognosis [4,15,16]. In this context, the application of the updated 2021 PNS-Care Score criteria has standardized patient classification, assigning fundamental weight to functional biomarkers [1,13]. 18F-fluorodeoxyglucose (18F-FDG) PET/CT has emerged as a superior-sensitivity tool capable of mapping hippocampal hypermetabolism and cortical dysfunction (the “tiara sign”) at stages where MRI shows no structural damage [11,15,17].

Given the probability that this entity is underdiagnosed, we report a patient with classical HL whose clinical debut was a state of profound stupor mediated by Ophelia syndrome. The objective of this report is to analyze the utility of molecular imaging in the diagnostic algorithm for unexplained coma in the oncological patient and to underscore how early functional diagnosis enables directed immunotherapy, achieving reversal of synaptic symptoms before irreversible neuronal damage ensues [1,12,18].

## Case presentation

A 59-year-old male was admitted for evaluation of lymphoproliferative syndrome under the care of the hematology service. Staging studies demonstrated adenomegalies at the cervical, thoracic, and abdominal levels, alongside pancreatic infiltration. During the hospitalization, the patient developed respiratory symptoms with impaired oxygenation and alveolar occupancy on chest radiograph, consistent with hospital-acquired pneumonia, which resolved with appropriate management. Concurrently, a polyserositis syndrome was identified, evidenced by ascites and a pleural effusion of mixed characteristics, considered related to the infectious process.

Four days after resolution of the infectious episode, the patient presented an unexplained deterioration of consciousness progressing to profound stupor without cranial nerve deficits or clinical seizure activity. He was transferred to the intensive care unit (ICU) for strict neurological monitoring. An initial structural neuroimaging approach via brain MRI with contrast at 3 Tesla, including an epilepsy protocol, showed no signal alterations in the medial temporal lobes, no acute vascular events, and no leptomeningeal enhancement; only mild cortical involutive changes were noted.

A subsequent multimodal diagnostic workup was initiated to determine the etiology of the neurological decline (Table 1). Cerebrospinal fluid analysis was unremarkable for infection, showing a clear appearance, zero cells per cubic millimeter, a glucose level of 49 mg/dL, and a protein level of 68 mg/dL. Flow cytometry detected no clonal B-cell or immature myeloid populations, and multiplex PCR for 14 neurotropic pathogens was negative. Metabolic and nutritional parameters were within normal limits, effectively excluding hepatic encephalopathy and Wernicke-Korsakoff syndrome. Systemic autoimmunity testing was negative for comprehensive panels, with the only finding being a reactive antinuclear antibody at 1:80 with a speckled pattern.

**Table 1:** Special diagnostic studies. * FilmArray meningitis/encephalitis panel (BioFire Diagnostics, Salt Lake City, UT): *Escherichia coli K1*, *Haemophilus influenzae*, *Listeria monocytogenes*, *Neisseria meningitidis*, *Streptococcus agalactiae*, *Streptococcus pneumoniae*, *Cytomegalovirus*, *Enterovirus*, herpes simplex virus 1 and 2, human herpesvirus 6, human parechovirus, varicella-zoster virus, and *Cryptococcus neoformans*/*gattii*. † Neuronal IgG antibodies tested: titin, SOX1, recoverin, Hu, Yo, Ri, PNMA2 (Ma2/Ta), CV2, and amphiphysin. ‡ Includes anti-Sm, anti-RNP, anti-Ro/SSA, anti-La/SSB, anti-Jo-1, anticardiolipin IgG/IgM, anti-smooth muscle antibody, antimitochondrial antibodies, and the antiphospholipid panel (beta-2 glycoprotein I, cardiolipin, phosphatidylserine, phosphatidylinositol, phosphatidic acid). Positivity cutoffs: ENA/cardiolipin antibodies ≥ 20 RU/mL; anti-smooth muscle > 30 U; antimitochondrial ≥ 10 IU/mL; antiphospholipid panel > 10 U GPL/mL each. ADA: adenosine deaminase; ANA: antinuclear antibodies; ANCA: antineutrophil cytoplasmic antibodies; CSF: cerebrospinal fluid; EIA: enzyme immunoassay; ENA: extractable nuclear antigen; IFA: indirect immunofluorescence assay; KOH: potassium hydroxide; NMDA: N-methyl-D-aspartate; RU: relative units; TSH: thyroid-stimulating hormone.

Diagnostic category	Parameter evaluated	Result	Reference value/Interpretation
Thyroid panel	TSH	3.64 µIU/mL	0.35 - 4.94 µIU/mL (normal)
	Free T4	0.79 ng/dL	0.7 - 1.48 ng/dL (normal)
Metabolic and nutritional	Ammonia	24.8 µg/dL	19 - 54 µg/dL (normal)
	Vitamin B1 (thiamine)	13.8 mmol/L	8 - 30 mmol/L (normal)
	Vitamin B12	848 pg/mL	239 - 931 pg/mL (normal)
	1,25-dihydroxy vitamin D	87.4 pg/mL	18 - 72 pg/mL (elevated)
CSF – Cytochemistry	Appearance	Clear, transparent	Clear (normal)
	Clot	Not present	Absent (normal)
	Xanthochromia	Negative	Negative (normal)
	Cell count	0 cells/mm³	0 - 5 cells/mm³ (normal)
	Red blood cells	0 cells/mm³	0 cells/mm³ (normal)
	Glucose	49 mg/dL	40 - 70 mg/dL (normal)
	Protein	68 mg/dL	15 - 45 mg/dL (mildly elevated)
CSF – Microbiology and immunology	Culture	Negative at 5 days of incubation	Negative
	Direct exam (KOH)	No fungal structures observed	Negative
	India ink	Negative	Negative
	Cryptococcal antigen (immunochromatography)	Negative	Negative
	ADA	1.1 U/L	Up to 5 U/L in CSF (normal)
	FilmArray Meningitis/Encephalitis panel (14 pathogens)*	All negative	Negative
	CSF flow cytometry	0.28% mature T lymphocytes/µL; 0.12% monocytes/µL; no immature B-cell or myeloid population	No clonal populations (normal)
Autoimmune profile	Anti-NMDA receptor (IFA) and paraneoplastic neuronal antibody panel†	All negative	Negative
	ANA (IFA)	Reactive 1:80, speckled pattern	Healthy population: 1:40 (20-30%), 1:80 (10-15%), 1:160 (≤5%), 1:320 (≤2%)
	Anti-dsDNA (IFA)	Non-reactive	Significant titer ≥ 1:10
	ANCA (c- and p-patterns)	Negative	Significant titer ≥ 1:20
	Extended ENA, organ-specific and antiphospholipid panel‡	All negative	Negative (assay-specific cutoffs in footnote)

Five days after ICU admission, the patient developed progressive acute kidney injury requiring alternate-day renal replacement therapy, without fulfilling urgent dialysis criteria. An empirical therapeutic trial with levetiracetam for suspected non-convulsive status epilepticus yielded no clinical response. Given persistent stupor and exhaustive exclusion of infectious, metabolic, toxicological, structural, and systemic autoimmune etiologies, paraneoplastic encephalopathy was strongly suspected (Table 1).

**Figure 1:**
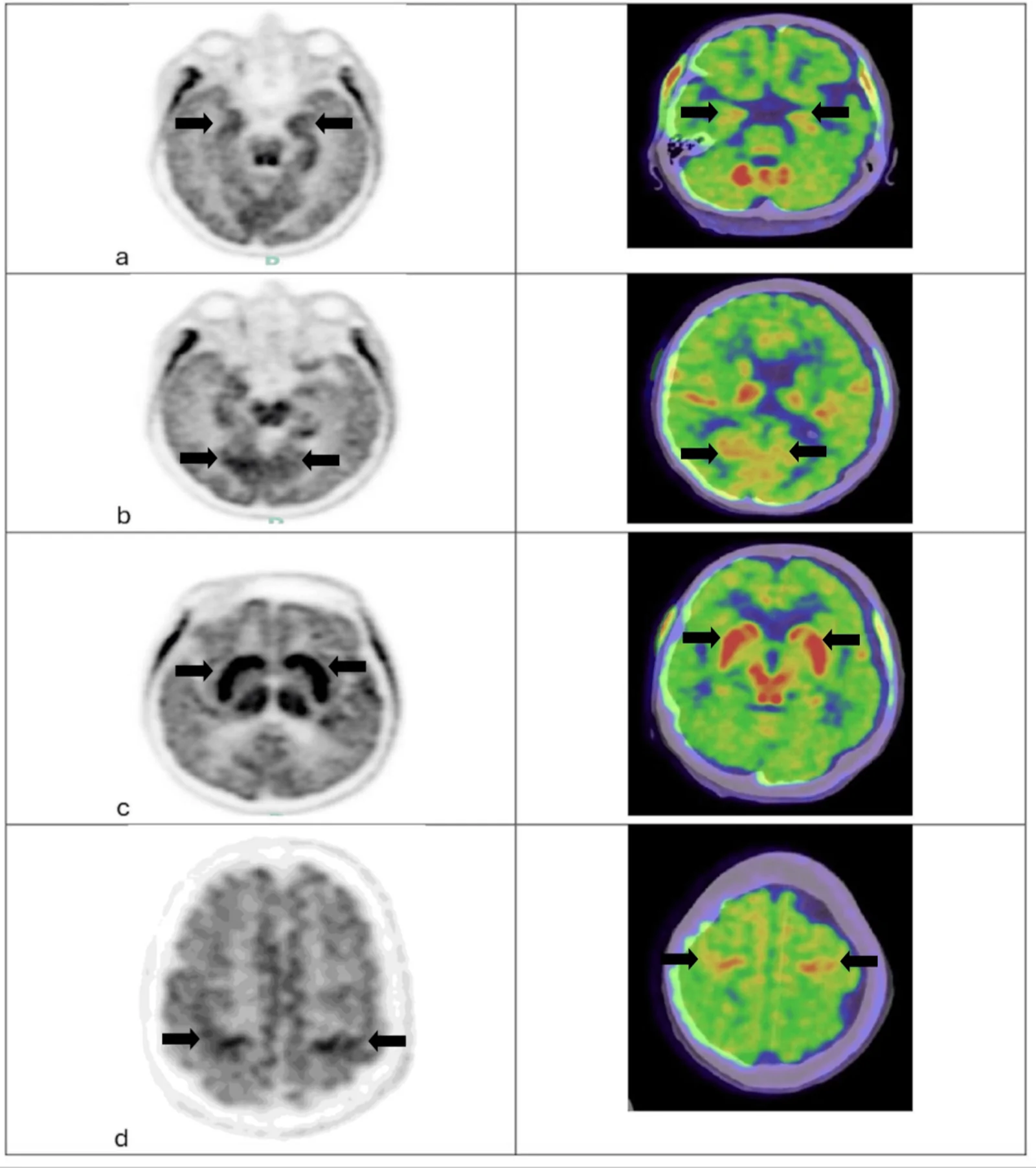
Brain 18F-FDG positron emission tomography/computed tomography (PET/CT). (a) Marked bilateral hippocampal hypermetabolism (black arrows). (b) Diffuse cortical hypometabolism with relative metabolic preservation of the primary visual cortex (black arrows). (c) Bilateral hypermetabolism of the basal ganglia (black arrows). (d) Diffuse cortical hypometabolism with relative metabolic preservation of the primary motor cortex (black arrows). Together, these findings are indicative of active synaptic neuroinflammation (a) and reflect the "tiara sign" (d), suggesting profound thalamocortical disconnection in the setting of paraneoplastic limbic encephalitis. 18F-FDG: 18F-fluorodeoxyglucose.

An 18F-FDG PET/CT of the brain was performed (Figure 1). The study revealed marked bilateral hippocampal hypermetabolism, serving as a marker of active synaptic neuroinflammation. Furthermore, diffuse cortical hypometabolism was observed with relative metabolic preservation of the primary motor and visual cortices (the “tiara sign”), suggesting profound thalamocortical disconnection. Hypermetabolism of the basal ganglia was also noted. These findings are highly consistent with paraneoplastic limbic encephalitis.

Fifteen days after ICU admission, histopathological examination of a lymph node and spleen biopsy confirmed the diagnosis of mixed-cellularity classical HL. Integrating the clinical phenotype (two points), the association with HL (three points), and the functional PET/CT positivity (two points), the patient was classified as a definite paraneoplastic neurological syndrome with a PNS-Care Score of 7. The diagnosis of Ophelia syndrome was established, considered most likely mediated by anti-mGluR5 antibodies, although specific antibody testing was not available at the institution.

**Figure 2:**
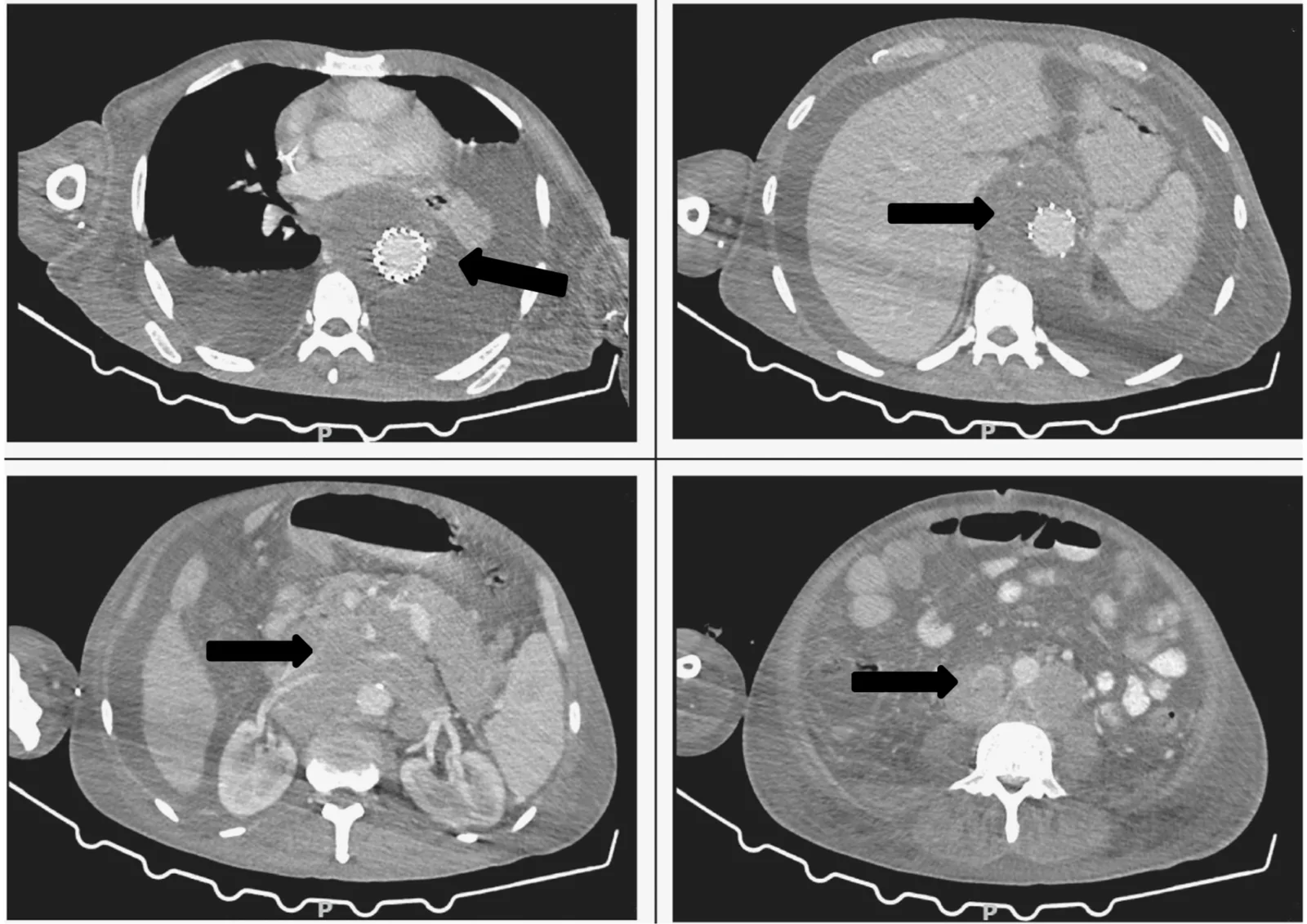
Contrast-enhanced abdominal computed tomography (CT). Axial views demonstrating a bulky retroperitoneal lymph node conglomerate encasing major abdominal vessels, alongside focal splenic and pancreatic infiltration. These findings correspond to the advanced underlying classical Hodgkin lymphoma driving the paraneoplastic immune response.

A contrast-enhanced abdominal CT (Figure 2) demonstrated a bulky retroperitoneal lymph node conglomerate encasing major abdominal vessels, alongside focal splenic and pancreatic infiltration, corresponding to the extent of the underlying classical HL driving the paraneoplastic immune response.

Based on these findings, rescue immunosuppression with methylprednisolone pulses (1 gram per day for three days) was initiated. Within 72 hours, the patient dramatically recovered wakefulness and the ability to follow complex commands. He subsequently started first-line ABVD (doxorubicin, bleomycin, vinblastine, and dacarbazine) chemotherapy, achieving complete resolution of the neurological syndrome.

## Discussion

This case illustrates the complex interaction between oncological disease, paraneoplastic manifestations, and comorbidities in a critically ill patient. Ophelia syndrome is an immune-mediated paraneoplastic manifestation of HL driven by anti-mGluR5 antibodies [5,10], characterized by a diverse spectrum that may include hallucinations, behavioral changes, cognitive dysfunction, seizures, movement disorders, sleep disturbances, and, as in this case, impaired consciousness progressing to stupor [5].

The sensitivity of MRI in surface-antibody-mediated encephalitis ranges between 50% and 70% [3,16]. By contrast, 18F-FDG PET/CT of the brain acts as a biomarker of synaptic dysfunction and microglial activation that precedes visible structural changes [11]. The observed pattern of bilateral hippocampal hypermetabolism is characteristic of the acute phase of mGluR5-mediated PLE [2,14], while diffuse cortical hypometabolism is a functional correlate of the severity of consciousness impairment [17]. Integration of PET/CT into the diagnostic algorithm for oncological encephalopathies significantly reduces the time to definitive diagnosis [1,15].

HL is distinguished by a unique immune architecture. Hodgkin and Reed-Sternberg (HRS) cells frequently harbor alterations in chromosome 9p24.1, leading to overexpression of PD-L1 and PD-L2 ligands and facilitating evasion of cellular immune surveillance [6]. Paradoxically, this aberrant inflammatory microenvironment and the ectopic expression of mGluR5 by HRS cells act as the trigger for B-cell self-tolerance breakdown [10,15]. Unlike PNS associated with intracellular onconeural antibodies (e.g., anti-Hu), where T-cell-mediated cytotoxicity generates irreversible neuronal loss, anti-mGluR5 antibodies are pathogenic through direct binding to extracellular epitopes [5,18]. This mechanistic distinction explains the remarkable clinical reversibility observed in this case: suppression of the humoral response and reduction of tumor antigenic load allowed restoration of glutamatergic signaling without residual structural damage.

## Conclusions

Ophelia syndrome represents a neuro-oncological emergency with a narrow therapeutic window. This case demonstrates that a normal brain MRI does not exclude paraneoplastic limbic encephalitis and that impaired consciousness, including profound stupor, can be its primary clinical manifestation. When unexplained stupor or coma occurs in an oncological patient with an otherwise non-diagnostic conventional workup, 18F-FDG PET/CT should be incorporated early into the diagnostic algorithm. Its characteristic functional metabolic signature, including bilateral hippocampal hypermetabolism, the tiara sign, and basal ganglia involvement, enables timely PNS-Care Score classification and prompt initiation of immunosuppressive therapy. Early immunosuppression, combined with oncological treatment targeting the underlying neoplasm, can achieve complete neurological recovery before irreversible synaptic damage ensues.

Recognition of this reversible and potentially life-threatening syndrome requires a high index of clinical suspicion and multidisciplinary collaboration between intensivists, hematologists, neurologists, and nuclear medicine specialists.
